# What constitutes good ethical practice in genomic research in Africa? Perspectives of participants in a genomic research study in Uganda

**DOI:** 10.1080/11287462.2019.1592867

**Published:** 2019-03-24

**Authors:** Rwamahe Rutakumwa, Jantina de Vries, Michael Parker, Paulina Tindana, Oliver Mweemba, Janet Seeley

**Affiliations:** a Medical Research Council/Uganda Virus Research Institute and London School of Hygiene and Tropical Medicine Uganda Research Unit, Entebbe, Uganda; b Faculty of Health Sciences, University of Cape Town, Cape Town, South Africa; c Nuffield Department of Population Health, Wellcome Centre for Ethics and Humanities (Ethox), University of Oxford, Oxford, UK; d Navrongo Health Research Centre, Navrongo, Ghana; e Department of Health Promotion & Education, School of Public Health, University of Zambia, Lusaka, Zambia

**Keywords:** Consent, feedback, genomic research, Africa, stakeholder engagement

## Abstract

Previous research has consistently highlighted the importance of stakeholder engagement in identifying and developing solutions to ethical challenges in genomic research, especially in Africa where such research is relatively new. In this paper, we examine what constitutes good ethical practice in research, from the perspectives of genomic research participants in Uganda. Our study was part of a multi-site qualitative study exploring these issues in Uganda, Ghana and Zambia. We purposively sampled various stakeholders including genomic research participants, researchers, research ethics committee members, policy makers and community members. This paper presents the findings from in-depth interviews with 27 people with diabetes who had participated in a diabetes genomic study. Data were collected using semi-structured interviews. Manual thematic content analysis was conducted using a framework approach. Findings indicate three key requirements that research participants see as vital for genomic research to be more responsive to research participants’ needs and contextual realities: (1) de-emphasising the role of experts and institutions in the consenting process, (2) clarity about the timing and nature of feedback both of findings relevant to the health of individuals and about the broad progress of the study, and (3) more effective support for research participants during and after the study.

## Background

Genomic research raises key ethical challenges related to consent, privacy, ownership of samples and data sharing (Cambon-Thomsen, Rial-Sebbag, & Knoppers, 2007). Questions have also been raised about how to explain key scientific concepts in lay terms to improve research understanding (de Vries et al., 2011; Nyika, 2009; Tindana et al., 2012; Upshur, Lavery, & Tindana, 2007; Wonkam, Kenfack, Muna, & Ouwe-Missi-Oukem-Boyer, 2011). Several studies have emphasised the importance of community engagement as a means of facilitating participants’ understanding of the research (Ahmed & Palermo, 2010; Boutin-Foster et al., 2008). However, there is a dearth of empirical evidence particularly from the African continent that explores these and related challenges (Munung et al., 2015). For instance, it has been observed that what constitutes an effective community engagement model when conducting genomic research in Africa remains unclear (Tindana et al., 2015). The development of such a model necessarily requires eliciting the views of genomic research participants in the African context. Similarly, how best to explain key concepts in genomic research – for instance, those relating to data and sample sharing – may also require the input of a range of stakeholders. In addition, there is evidence that the challenge of making these concepts easily understood by research participants might also be compounded by some clinical research practices that appear to be common in some settings. For instance, a study conducted in North America revealed that only a small number of clinical researchers incorporate information about data sharing in their informed consent forms, because data sharing is not their responsibility (McGuire, Hamilton, Lunstroth, McCullough, & Goldman, 2008). There is a need to investigate the potential for this kind of issue to arise in the African setting, and to suggest mechanisms for ensuring awareness of the need for the inclusion of all essential information in consent documents.

Considering these gaps, researchers have highlighted the need to develop novel approaches to the informed consent process in the African context (Munung et al., 2015; Wright, Koornhof, Adeyemo, & Tiffin, 2013), and as some have noted such efforts would ideally incorporate input from multiple stakeholders including genomic research participants (Munung et al., 2015). The need to explore these approaches is particularly compelling in the case of Africa where genomic research is relatively new. A particularly important ethical issue identified in the literature has concerned whether researchers have a responsibility to feedback findings to participant, and if so what the nature and scope of those responsibilities might be (Johnson & Gehlert, 2014; Marsh et al., 2013). Carrieri et al. (2016) point out that participants’ preferences in relation to receipt of primary and incidental findings will differ from person to person and over time, and that caution needs to be exercised to avoid universal consent models that are insensitive to peculiarities of a given setting. While feedback of results remains a global issue, further research in Africa is particularly important given the rapid growth of genomic research on the continent compounded by factors such as low literacy, and cultural values and beliefs.

In this paper, we present the findings of a study we conducted to inform the development of a robust and supportive ethical and governance framework for genomic research in Africa. Our purpose in this research was to explore perspectives on good ethical practice in the conduct of genomic research from the point of view of those who have participated in a genomic study in Uganda.

## Methods

### Study design

We conducted a multi-site qualitative study in Uganda, Ghana and Zambia. At the Ugandan site, we purposively selected 27 individuals from a group of patients attending the same diabetes clinic and who had participated in a previous diabetes genomic study. These were carefully selected to ensure that only those who had participated in a genomic study at least six months earlier and, therefore, with sufficient experience of genomic research participation to draw upon were interviewed. The other stakeholders included two members of a research ethics committee (one of whom was a community advisory board member), six researchers and their team members (including four medical doctors, a research nurse and a counsellor), one policy maker, four community leaders (including two officials of the local administration system and two religious leaders) and five non-participants. Using semi-structured topic guides, in-depth interviews were conducted with these participants. This paper is based exclusively on data from interviews with 27 participants from the Ugandan site who had participated in a genomic study.

### Data management and analysis

All the interviews were voice-recorded – following consent from the interviewees – and transcribed. The interviews we used for the analysis presented in this paper were conducted in Luganda and were simultaneously transcribed and translated into English by trained translators. Manual thematic content analysis was conducted using a framework approach (Gale, Heath, Cameron, Rashid, & Redwood, 2013). This approach allows for the in-depth investigation of qualitative data by combining both case and theme-based analytical strategies (Smith & Firth, 2011). We used the framework approach because it is well suited for analysis involving multiple researchers and sites, as the case was with this study. The approach was particularly useful for our analysis as it provides a structure that facilitates a transparent process of analysing data by case and by code (Gale et al., 2013).

Ethical approval for this study was obtained from the Uganda Virus Research Institute Research Ethics Committee (SS 3856), and from Oxford University (OxTREC Reference: 580-16). All interviewees were assigned a unique identifier (an ID number) which was used on all transcripts. Real personal names and names of places of work or residence were not included in any transcripts. A database linking real names and ID numbers was stored securely and separately from the data and is accessible only to the PIs.

## Findings

Reflecting on their experiences as participants in a previous genomic study on diabetes, which was complemented by a number of clinical tests – discussed in more details below – our interviewees highlighted a number of research practices that they felt could be improved upon and suggested new ones that could be adopted as a way of making research more responsive to the participants’ needs and contextual realities. Their perspectives were wide-ranging, touching on participant recruitment and consenting practices, the conduct of the study and participant expectations from researchers, and what happens when the study ends. Our analysis suggested three particularly important themes: (1) information delivery and the consenting process, (2) feedback to research participants and (3) support during and after the study.

1. Information delivery and the consenting process

One of the key areas of concern for research participants was how information about the genomic project was delivered to them, and how informed their consent was for participating in the research. When asked about the genomic study in which they had participated 6–12 months back, 11 of the 27 participants initially reported that they were not aware that they had participated in any such study, implying that they thought procedures they went through – such as blood draws – were part of routine care at the hospital. On further reflection, interviewees did recall their research participation and the consent process. However, while they remembered they had participated in the study they also indicated that they had agreed to participate without a full and complete understanding of what this participation entailed. While less than perfectly informed consent may not necessarily be unique to genomic studies, it was clear that in this study a significant factor in this was the difficulty which participants found in understanding genomic research, as illustrated by the inaccuracies that were evident in their perceptions of genomic concepts. It is important to note that inadequately informed consent does not necessarily imply researchers’ failure to convey the study information fully to the participants. It was apparent in our interviews that participants did not seek clarification on issues or ask questions even when they were given ample opportunity (we return to this issue in more detail later), or they might have not fully grasped genomic concepts even after the researchers made efforts to explain these. Other explanations are that the participants possibly had forgotten about the study due to passage of time, or might have not been telling us the whole truth.

When asked how these information and consent challenges might be overcome, participants identified specific standard procedures that they felt would constitute good research practice. One such procedure related to delivery of study information to prospective participants. An issue of particular interest to participants was the context in which the study information is delivered and who should deliver the information. Participants highlighted the need for researchers to reach out to prospective participants within a community – rather than a health facility – context. They proposed that such delivery of study information involve community members as principal players, as presented in more detail later.

Our analysis yielded three key explanations for the choice of community as the context for information delivery and for engaging community members. First, it was thought that the community context would provide an environment for free interaction between researchers and prospective participants, where the latter would feel less inhibited to ask questions or raise concerns. This is especially so when individuals with whom community members identify are engaged in the information delivery effort. Such an environment contrasts sharply with that in a health facility context where power dynamics between the researcher/health worker and the participant/patient might influence information exchange and comprehension. The influence of this power relationship was evident in the perspectives of different participants on the power and authority of health workers. For some of these, asking a health worker for clarification amounted to challenging the authority of the health worker, as illustrated in an excerpt from an interview with Research Participant 26:

Interviewer: Now, when you see these blood samples being drawn from your body; how do you understand that?

Participant: I don't understand it … .

Interviewer: At times you have to ask. Now when they are bleeding and you do not ask, what are you up to?!

Participant: Ahh … , to ask a musawo [health worker] such a question! It is hard!

Similarly, advising a researcher/health worker on good practice in research was considered by some as a redundant exercise since these are trusted experts in their subject area, as echoed during an interview with Research Participant 20:

Interviewer: So what advice would you give us [on good practice in genomic research]?

Participant: My friend, what idea to give you; to such learned ones. …  [laughing a bit].

Interviewer: Oh yes! You can also have an idea to give us about anything … . What advice would you give us?

Participant: The learned ones know how to handle their work with their experience. I cannot give any idea  …  and they know better how to handle everything.

The fact that participants’ perception of unequal power relations between health worker and patient undermines their ability to ask questions and make suggestions may indicate that a health facility is a less-than-ideal context for obtaining informed consent, particularly when the researcher/health worker is the one interfacing with the prospective participant/patient in the consenting process. Notably, this may still be true when the researcher/health worker has made every effort to fully inform the participant about the study.

Indeed, it was evident in the above excerpts that the unequal power relationship between the researcher and the participant partly explains why participants in this study initially reported that they were not aware of their participation in the study – or indeed any study – and whose understanding and awareness was rather superficial once they had recollected their participation.

Interviewer: Can you please tell me about the genomic research project you participated in?

Participant: The research that I joined, we came here to the diabetes clinic and the medical staff explained to us that there is research that was going to start here to find out why diabetes is so rampant so as to find the drug for us.

Interviewer: Is that all that you were told; did they mention about genomic research?

Participant: The way they asked us the questions, was leading to genomic issues. Because they used to ask about my parents … .

Second, participants preferred their community as the context for information delivery because it was not enough to target only prospective research participants in delivering the study information. Based on these views, good ethical practice entailed an obligation not just to the participants but to the entire community from which the participants are drawn. The participants held the view that a new research project should serve as an opportunity for both the prospective participants and the entire community to learn about the health condition under study, and to be able to use this knowledge to take preventive or remedial action. Reflecting on the ideal strategy for information delivery about the study, one participant observed:

[Information delivery] would be like collecting people in the community and teaching them, examining them, bleeding them for samples and conducting sessions for all the people in the community to be informed about diabetes. Even those who have not acquired the disease, to be aware of it … . For example, you mobilise the community using the loud speakers, advising them to converge at Freedom City [mall] like they do for Agriculture and Veterinary. (Research Participant 09)

Third, participants preferred community members – rather than “experts” – as agents for information delivery because these were accessible both in terms of physical proximity, and fellow community members being able to identify with them and more likely understand and trust them. Community members were therefore believed to be more effective in articulating information about research in the community. According to the participants, these community members – who would preferably include officials from different entities such as local councils and community-based organisations, as well as religious leaders, women leaders and family heads – should constitute village committees in charge of information delivery. In suggesting this, participants seemed to highlight the importance of using the existing grassroots structures, including local government (Local Councils), and opinion leadership. As the following excerpt illustrates, some of them appeared to suggest that in the event of a new study, community members could be identified and sensitised about the proposed study, so that these can in turn play an instrumental role in the delivery of study information.

I think even at the village level, there should be the committee that would teach the grassroots people so that they also get well informed. So the committee would start from the village level or parish level. Because they [community members] deliver the information very well to the grassroots people. But when we have committees at the top, at the higher level  …  the information remains there; it does not reach the grassroots people.  …  (Research Participant 04)

It is noteworthy that this participant echoes a proposal, highlighted earlier, for information delivery efforts that target the broader community rather than only prospective participants. But study participants also identified some personal strengths or characteristics of community members that they considered ideal for the role of information delivery, while noting the need to build on these strengths by training them so they are in a better position to perform this role and also handle those in the community who are in need of help.

Religious leaders are very good at counselling, then the teachers, [they] pass on the information [easily] to others  …  then elders and mothers  …  in the community, they are more recognised and trusted. (Research Participant 08)

Community leaders should be trained in how to handle and sensitise their people. To reach that point they will have studied what should be done like how to handle HIV/AIDS patients. (Research Participant 01)

A rather different but related issue was the ongoing need for information and other forms of support to participants during the study. Against the background of having consented to participate with insufficient understanding of the study, some participants expressed concerns about unmet informational and emotional support needs during their participation. They looked to this support as a means of plugging the outstanding information gaps in their knowledge of the study they were participating in. For these participants, good ethical practice in this kind of research would require that researchers maintain regular dialogue with their participants, so the latter can gain a full understanding of the study. Regular dialogue would also comfort and assure the participants to allay some of their worries, including that their samples would be used for the purposes for which they were expressly intended.

[My advice would be that] when you draw the samples from us, you continue teaching and counselling us that “do not get worried, we are going to test this blood.” But we left there when we had not yet understood anything; I didn't understand anything … . I would suggest that when you come to us, you sensitize us about the issue … . Like for this blood that was bled from us recently, we remained wondering at that much blood they drew from us. So they took our blood and we said “what to do?” So we came back home just like that … . (Research Participant 27)

Yet other participants proposed ongoing information not because they were insufficiently informed when consenting to participate in the study, but because they wanted to be generally updated on new developments pertaining to their health condition, such as new therapies or dose prescriptions.

The recommendation I am giving is just to keep in touch with the patients [who donated samples]; whatever new thing that you discover, [you need] to keep us informed … . Yes, to keep in touch [with your patients], at least calling them, or if there are any changes, to give them the feedback. (Research Participant 19)

2. Feedback to research participants

The second major theme arising out of the analysis of our interviews with participants, concerned the issue of feedback of results from both the study itself and the other, clinical, tests they were offered at the time of recruitment. The range of tests offered was significant, as is clear from this extract from the project's participant information sheet.

The tests on your blood will include those that look for diabetes, possible anaemia, high cholesterol and to see how well your kidneys and liver are working. We will also test for infectious diseases like HIV … .. If you wish, you can choose to receive some of your test results … . The results you can choose to collect are: HIV results, haemoglobin, HbA1C, Fasting plasma glucose, BMI and blood pressure. We will not provide the results of those tests related to your genes. This is because scientists do not yet fully understand the role of these genes in disease, and so the information may not be directly useful to you.

When our interviewees raised concerns about feedback this tended to take two forms: concern about delays and lack of clarity about timing, and views about how the feedback of information deemed sensitive should be handled. With regard to the first of these, several participants expressed disappointment over the perceived delay in receiving their results. Some seemed to speculate that the reason why the results were not being returned was that the researchers thought that participants were too scared of the possibility of the results indicating a serious health condition.

I for one, I need my data [results]  … . I will not fear anything, be it HIV … . So there is nothing to fear – the more you fear, the more you are sabotaging your own life … . What I feel much concerned about is to get my results … . (Research Participant 02)

Participants narrated their experiences of delayed results, and what the delay meant to them. For the majority, they considered that delayed results meant that they were not able to effectively track the progress of their illness and make informed decisions around lifestyle adjustments and the right medication and dosage, given their health condition and its gravity.

My issue is, I want to know my results, let them tell me what type of Diabetes I have so that I get the medicine that can sustain me well, because they will have tested my blood well … . [Researchers need to] inform each participant about research results [in order to answer questions such as] “In what group of diabetes is that person”? I’m so much concerned about that. (Research Participant 09)

Give us the results so that we get to know whether diabetes [blood sugar] has reduced. Tell us where you have reached so far. In case of any change in my health, [the knowledge of my results] would ease follow up to improve my health … . (Research Participant 10)

For others, the implication of delayed results was more serious, in that the longer it took to get their results back, the closer they got to a point at which it would be too late to benefit from the available therapy.

I happen to think about the blood sample results that have really taken long. When shall we ever know the results? Could be by that time some of us will have already died! (Research Participant 13)

Yet for the other participants, getting their results back and on time seemed to be the minimum expectation from their voluntary participation in the study. These implied that it was their right to be informed about their results given what some perceived as the sacrifice they made in offering their blood sample.

The issue [I would like to stress] is to give us the results … . Let them show us the results … , explain to us where to go for treatment, what drug to use. It is so hurtful when you have participated in the research and you don't get the outcome of the research. (Research Participant 08)

Research Participant 11 did not express anticipation of results from researchers but only suggested the researchers’ return of the results was the correct thing to do:

You [the researchers] have our phone numbers. You can always call us back at any time when you need us, because we would also wish to know the outcome.

We infer based on these participants’ narratives that good ethical practice on the part of the researcher calls for a clearly laid out framework for the return of individualised research results, especially if the results contain information with major implications for the participant's health. In this particular study, the questions of feedback related to the clinical tests offered complementary to the genomic research – it was clear that there would be no feedback of genomic findings. However, our findings do suggest both, on the one hand that when feedback of any kind is offered it is vital that there is clarity about what can be expected and that these expectations are in fact met. And, on the other hand, that there is a broad expectation that clinically useful information should be fed back. Participants seemed unaware of the fact that genetic results might sometimes fall into this category, but the broad implication of their comments is that careful consideration ought to be given to feedback within research studies taking place in the clinical setting.

It is noteworthy that the perspectives on feedback presented above, relate primarily to individualised rather than generic feedback i.e. to feedback relevant to the participant's own health. In addition to this, however, some participants expressed an interest in more general feedback about the progress of the research and about its findings is an important aspect of the researchers’ responsibility to participants and communities. This was expressed by Research Participant 18 who did not indicate the expectation of researchers returning his test results but did highlight the importance of generic feedback when prompted to suggest how better genomic research can be conducted and managed: “because you took our blood samples, you should always call us back as the witnesses of the research.” The same participant went on to suggest the institutionalisation of the researchers’ return of results to participants:

Interviewer: In your view, should there be specific policies governing genomic research?

Participant: Yes.

Interviewer: What are your views?

Participant: Like teaching us, even telling us what they have found in our blood, and keeping us updated.

The other aspect concerning the issue of feedback was the question of how to deliver sensitive results. Participants discussed potential sensitivities about the results generated from their samples, and the way they are fed back to sample donors. It appeared from our data that they were more particularly concerned about incidental results that were not necessarily related to the original purpose of the tests. They noted the likely family fallout that could ensue if caution was not taken in the delivery of such feedback. In the light of these concerns, our findings suggest that participants did not take for granted the researchers’ commitment to confidentiality as spelt out in the study information sheets. For these participants, good ethical practice in the provision of feedback therefore necessitated the strict upholding of confidentiality but also a clearly laid out procedure for disclosure of sensitive results to third parties if the participant, not the researcher, approved of this.

It [result] should be confidential, in case it is found out that it is such a scaring illness that could scare the husband, so as not to disclose to him. Or else  …  counsel the husband seriously then disclose to him otherwise this can easily break the marriage. For example we have come to participate in the research about diabetes, then the test shows that there is HIV as well. Let them tell you [sample donor] first, [followed by] serious counselling before calling the husband  …  because I have come alone and I was invited as [sample donor] but not as Mrs. so and so. (Research Participant 08)

3. Support during and after the study

Reflecting on their experience of participating in a previous genomic study, a significant number of participants cited insufficient or lack of support from the researchers as a key issue that needed to be addressed. These sentiments were reiterated during a dissemination event for the research participants: during question time, most of the concerns and questions raised by the participants were to do with the perceived gaps in diabetes care and the urgent need for help in addressing these. We view this as an ethical challenge pertaining to the conventional practice of clinical research in general, not just genomic research.

There were several aspects to the perceived insufficient or lack of support from the researchers. For a minority, it was about the compensation they received for participating in the study, which they considered less than they deserved. Some of them interpreted this as a lack of recognition of their generosity in donating their samples for research.

My issue in this research is, we are given very little money for transport, plus feeding, at least they would give us 30,000/= otherwise 15000/= was so little [given that] one would be bled for 6 vacutainers of blood sample … . For a person to consent for a genomic study, and donate samples for the research! (Research Participant 11)

In case this blood sample has helped you and when you still have the participant in your records, then you would be considerate and give a token to those that participated in the research … . (Research Participant 10)

For other participants, the concern was about the absence of support for participants who could no longer travel to health facilities to obtain care due to a deterioration in their health. They argued that it was premature for the researcher-participant relationship to end once samples were drawn. In this case, good ethical practice according to these participants required sustained contact between the researcher and the participant/patient. For some, sustained contact would particularly include home visitations to follow up on former participants.

I would suggest that there be a policy to go to sick people's homes to find more about their genomic health; for those who cannot come here to the hospital for treatment or examination … . I suggest that it be an ethical requirement to] have people who are responsible for the [outreach] exercise. (Research Participant 23)

[We prefer that] researchers do not neglect us patients; like now we have joined the research, and after a while we do not hear about the research any more. When they have already taken our blood samples and thereafter we lose the follow up. So let this contact continue; this would be so helpful to us. (Research Participant 22)

A more frequently raised concern about support to research participants related to prescription drug procurement. Participants narrated the difficulties they encountered in ensuring that they had a constant supply of the required prescription drugs, because of the prohibitive cost of the drugs. These participants were unequivocal in their appeal for help in procuring the drugs either by way of full financial cover or discounted prices.

My recommendation would be to reduce drug prices or to go about it like they did for HIV/AIDS [i.e. free ART]. At least when the drug is at a reasonable price. Now this [diabetes] drug is for one month, when you add the consultation fee, plus this Shillings90,000/= that I pay when I come every month, where to get the money then?! (Research Participant 14)

We are charged a lot of money! And when we are seated [in the doctor's waiting room] with other patients you can hear some of them expressing similar sentiments. One of them noted: “I will not report this other health problem because if I do so they will write more drugs, when I do not have the money, and that will make me worry so much which may worsen my health condition.” Sincerely you notice some people who cannot afford just die so fast. So I would appeal to be helped like it is done for HIV patients who are given free drugs. (Research Participant 01)

But the excerpt from the interview with Research Participant 01 above also highlights the complexity of inaccessible prescription drugs. As the participant suggests, the prohibitive cost of drugs did not just make it difficult for patients to access prescription refills for the treatment of a known condition. By causing the patients to refrain from revealing to the health worker new developments in their health condition for fear that this would otherwise lead to an additional prescription for which they were not able to pay, high drug costs also undermined patients’ full disclosure of health status.

Some of the participants lamented that they did not deserve the experience of not being in a position to procure the prescribed drugs given their advanced age and the substantial amount of money and time they had invested over the years in the facilities where they sought healthcare.

I have already told you that we, the aged ones, no longer have money to buy the drug. There could be an arrangement for us to be supported with the drug or to reduce the prices of the drug; we have really spent long here and we have put in a lot of money at this hospital for the treatment. Right now when you do not come with Shillings150,000/= to 200,000/= you cannot get the drug to sustain you for a month! … . Like for me I have many illnesses; arthritis, ENT problem, Ulcers, Diabetes, hypertension  …  . That is the biggest problem for us the aged ones, and you cannot do anything for income generation. We have to feed, we do not have any support and our children have their own problems and responsibilities. (Research Participant 15)

Musawo [health worker], my appeal is to be helped with some reasonable amount of drug covering like a month or two to sustain us, but they give us very little drug and tell us to come back in a month when we do not have money. The drug is quite expensive! (Research Participant 27)

Others suggested that they deserved support from researchers because of their voluntary participation in the study. They noted that they could benefit from trial drugs, but also from associated support – such as food assistance. Such support, according to the participants, would facilitate proper day-to-day management of their medication for minimising its negative effects and enhancing their quality of life.

There is one issue I would like to talk about: the researchers should make a drug and give us as they try it while carrying out the research. … they give you the drug and draw samples from your body for the first time, then afterwards they come back and check to see whether the drug is effective or not, or even to help us …  some of us do not have any help from anywhere. The diabetes drug is so expensive, at least when you participate in the research and you are given the drug it can sustain you for a while  …  . Because at times when they go back to the participants they find the participants have already died  …  and at times some die just because they cannot afford the drug. That is why it is said that diabetes is for rich people  …  . [Also] biologically the drug we swallow can be poisonous in our body when you do not eat the food that can dilute the drug. Yet this food is so expensive, so they should support us so as to live longer to sustain our children till the time when we leave this world. (Research Participant 26)

[We would appreciate] if the researchers could help us and give us the drug  …  or get us items for use as we the diabetic participants. (Research Participant 18)

## Discussion

In this paper, we explore perspectives on good ethical practice in the conduct of genomic research from the point of view of those who have been involved as research participants in a genomic study in an African clinical context. Our analysis suggests the importance of three themes: (1) information delivery & the consenting process, (2) the need for clarity about what feedback can be expected and when and (3) support during and after the study. These findings suggest some enduring gaps in clinical research ethics in general but also more particularly in genomics and the need for further engagement and research on these issues. It was clear, for example, that the question as to how best to obtain adequately informed consent for genomic research in low literacy settings remains an important and unresolved challenge. In addressing this question, findings from previous studies in other countries (de Vries et al., 2011; Emerson, Singer, & Upshur, 2011; Tekola et al., 2009; Tindana et al., 2012; Tindana et al., 2015) have highlighted the importance of community engagement as a means of identifying processes that are culturally acceptable and that enhance prospective participants’ understanding of the study. Our study builds on this evidence base by shedding some light on Ugandan participants’ perspectives particularly on who should be involved in obtaining consent, and in which kinds of contexts the consent should be sought. Findings from this study point to the need to de-emphasise the role of experts (doctors and other health workers) and institutions (health facilities), and to instead principally engage community members and spaces as a means of obtaining adequately informed consent for research participation. Indeed, our findings suggest that, on reflection, our interviewees felt that they had consented to participate without adequate information about the study, which echoes findings from several previous studies (Robinson, Slashinski, Wang, Hilsenbeck, & McGuire, 2013; Traore et al., 2015).

In addition, the ongoing need for information and other forms of support to participants during the study – which was expressed by those who reported having not been adequately informed about the genomic study they had participated in – raises another important question: should information delivery be a one-off exercise to be conducted at the time of participant recruitment, or should it be a process that spans both the participant recruitment and study implementation phases? Our findings suggest participants’ view that in the same way that ideal consent has been viewed as a process (Kaye et al., 2015; Stein & Terry, 2013), study information delivery ought to be treated as a process in which researchers continue to interface regularly with participants to, among other things, clarify or reaffirm the study information already delivered and assure participants about the honesty and well-meaning intensions of the researchers. Study information delivery could therefore be viewed as ideally a process for building trust through establishing a long-term relationship between the researcher and participants.

Our findings suggest that the current, conventional ethical standards and practices may not be sufficiently responsive to participant and community concerns and interests in addressing the complexity of the ethical challenges in research. A good example of this is the expectations expressed by our interviewees about feedback of information to them personally and about the progress of the project more generally. Firstly, it is clear that where feedback of any kind is a possibility this must be explained clearly, and participants should be given reasonable expectations about the nature of any possible feedback and its likely timing. Secondly, although feedback of genomic findings was not an option in this study, participants broader views about the importance of feedback as part of the obligations of researchers suggest that where genetic or genomic findings of direct clinical relevance are a possibility these should be offered unless there are good reasons not to do so.

Our observations lend support to those by other scholars such as Bredenoord, Onland-Moret, and Van Delden (2011, p. 861) who provide similar and other grounds for the assertion that “valid reasons exist to adopt a duty to return genetic research results … .” Others have gone a step further by advocating a shift in the debate from whether or not to provide individualised results to finding an acceptable compromise between the benefits and harms of disclosing the results to participants (Bredenoord, Kroes, Cuppen, Parker, & van Delden, 2011).

Similarly, the participants’ recurrent expression of concern about their difficulty in procuring required drug prescriptions and their appeal to the researchers for help with drug support highlight yet another ethical gap, whereby the accepted ethical standards and practices seem to reflect the notion that such support, even when it is acutely needed by the participants, is beyond the purview of research ethics. But should the ethical principle of beneficence, which places an obligation on researchers to maximise possible benefits for research participation (Department of Health and Human Services, 1979), not be brought to bear in this case? Thus, should support in procuring drugs, at least in some form, not be considered a researcher's responsibility to the participant? We believe that these questions are legitimate and justify further consideration.

In summary, in this paper we identified a number of research practices that could be improved upon in order for clinical research to be more responsive to research participants’ needs and contextual realities. Specifically, we suggested changes in three thematic areas: (1) information delivery & the consenting process, (2) feedback to research participants and (3) support during and after the study.

As described, the empirical research reported in this paper forms part of a broader project involving stakeholders in Uganda, Ghana and Zambia. Papers from Ghana and Zambia will be published separately. The totality of our findings will be made available to the H3Africa ethics working group to help support them in their development of a robust and supportive ethical and governance framework for genomic research in Africa.
